# Neurologic monitoring in children and adolescents with hepatic failure: new mechanisms and non-invasive approach

**DOI:** 10.1016/j.clinsp.2025.100844

**Published:** 2025-11-25

**Authors:** Beatriz Kelly Oliveira Silva, Maria Clara Silveira de Carvalho, Artur Figueiredo Delgado, Werther Brunow de Carvalho, Michele Luglio

**Affiliations:** Pediatric Intensive Care Unit, Instituto da Criança e do Adolescente do Hospital das Clínicas da Faculdade de Medicina da Universidade de São Paulo (ICr/HCFMUSP), São Paulo, SP, Brazil

**Keywords:** Hepatic failure, Pediatric critical care, Intracranial hypertension, Neuromonitoring, Liver dysfunction

## Abstract

One of the main complications of liver failure, whether acute or chronic, is the progression to Hepatic Encephalopathy (HE), cerebral edema, and, consequently, intracranial hypertension. Several mechanisms explain the progression to HE, but the key point is the impairment of the hepatic metabolism of ammonia and other toxic metabolites, causing vasogenic and cytotoxic cerebral edema and alteration of cerebral autoregulation. In the pediatric population, the diagnosis of HE is more challenging due to the difficulties in neurologic assessment in this group. Thus, the role of multimodal assessment with several monitoring methods is increasingly more important. Among these methods, invasive monitoring of ICP is increasingly being discouraged, giving way to other noninvasive assessments such as methods of assessing intracranial pressure (Brain4Care®, optic nerve sheath diameter), cerebral blood flow (transcranial doppler), and cerebral SatO_2_ and O_2_ consumption (Near-Infrared Spectroscopy, Jugular venous oxygen saturation). Thus, the multimodal evaluation of these patients has been gaining strength for better diagnosis and management of neurological conditions in pediatric patients with liver failure.

## Highlights

• Non-invasive neurologic monitoring is feasible in pediatric ALF.
• Optic nerve sheath diameter detects intracranial hypertension in pediatric ALF.
• Multimodal, noninvasive tools guide better neurological pre-transplant assessment.

## Introduction

Liver diseases can result from either acute liver failure or chronic liver disease. Acute liver failure represents a hepatocellular necrosis in the absence of previous liver disease. Chronic liver disease results from prolonged and persistent liver damage leading to fibrosis and scarring, eventually culminating in cirrhosis. Another possible presentation is Acute-on-Chronic Liver Failure (ACLF), which is a multisystem organ failure syndrome with a phenotype like acute liver failure but in a patient with previous cirrhosis.^1^

In adults, Hepatic Encephalopathy (HE) is a cardinal finding for the diagnosis of acute liver failure. Minimal HE in adults can be seen only with neuropsychological and psychometric testing. When evident, the severity of HE is described by the progression of neurological findings graded by the West Haven scale. In children, HE is not uniform and, when present, may appear late in the course of the disease. In addition, the assessment of neurocognitive abnormalities is more difficult in this population.^2^

**Fig. 1:**
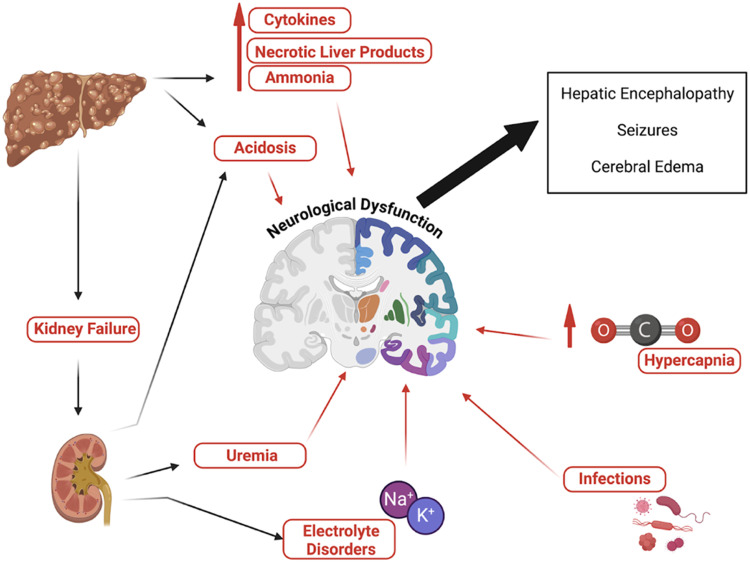
Mechanisms involved in liver failure neurological dysfunction.

Changes in neurological function in patients with liver disease are multifactorial and may include factors such as hypercapnia, acidosis, uremia, fluid and electrolyte disturbances, delirium, seizures, stroke, or Central Nervous System (CNS) infection (Fig. 1). Cerebral edema is a common finding in those with acute liver failure and ACLF.^3^

In the past, neurological evaluation in patients with acute liver failure and fulminant hepatitis was limited to serial neurological examinations. However, over time, this assessment proved to be insufficient, since brain abnormalities were found late and at irreversible stages.^2^ In recent years, the concept of multimodal neurological monitoring and neurophysiological support has emerged with various technologies that allow early diagnosis of hemodynamic, electrical, biochemical, and physiological brain alterations, leading to better survival rates.^4^

In pediatrics, this multimodal assessment is extremely important, since invasive monitoring poses a greater risk in this population and due to limited evidence on the positive impact of its use on prognosis. Thus, there is greater interest in the use of less invasive methods in children, such as transcranial Doppler, Near-Infrared Spectroscopy (NIRS), and optic nerve sheath diameter. Despite this, the evidence for the use of these methodologies in pediatrics is limited, with data from studies in adults being extrapolated. Therefore, clinical trials and more robust studies are increasingly needed to validate their use^2^ and application to the pediatric-specific setting still needs to be further evaluated.

### Epidemiology of cerebral edema in liver failure

The cerebral edema is a key feature of HE, but it is not synonymous with the condition.^1^ Cerebral edema and Intracranial Hypertension (ICH) are complications in approximately 75% to 80% of patients with fulminant hepatitis, accounting for a mortality rate of 20%‒35%.^3^^,^^5^ In patients with grade III HE, the frequency of ICH ranges from 25%‒35% and in grade IV, up to 75%. Other risk factors for ICH are female gender, younger age, severe liver failure (MELD > 32), ammonia above 150‒200 mcmoL/L and renal failure.^5^

The development of ICH associated with cerebral edema is more common in acute liver failure. In chronic liver failure, ICH is uncommon, occurring in approximately 5% of patients, which contrasts with magnetic resonance imaging studies that demonstrate the presence of cerebral edema in patients with compensated chronic liver disease, and the severity of HE may be related to the degree of edema.^6^, ^7^, ^8^ In critically ill patients, cerebral edema is equally present in acute liver failure and ACLF.^1^

In the pediatric population, the early stages of HE are difficult to assess, and cerebral edema associated with acute liver failure may not be apparent until later stages.^9^ Cerebral edema leading to ICH has been reported in up to 80% of children with severe acute liver failure and HE, and the major cause of death in children with acute liver failure who did not receive liver transplantation is multiorgan failure, with ICH accounting for 20%‒35% of deaths.^9^^,^^10^

### Pathophysiology of hepatic encephalopathy and cerebral edema

HE is a neuropsychiatric syndrome defined as cerebral dysfunction caused by liver failure and/or portosystemic shunt.^1^^,^^3^ Its presentation has a wide spectrum, from minimal neurological dysfunction to coma and death due to cerebral herniation.^11^ The key point in the evolution of HE is the reduced hepatic capacity for ammonia detoxification, which leads, through several pathways, to the formation of cerebral edema.^3^ Cerebral edema in liver failure occurs through three main mechanisms: vasogenic edema, cytotoxic edema, and cerebral hyperemia and the importance of each type of edema in the pathophysiology varies according to the type of hepatic injury.^1^ The central cell in the development of cerebral edema is the astrocyte, an electrically inactive multifunctional glial cells that regulate the ionic gradient, cerebral blood flow, integrity of the blood-brain barrier, reactive gliosis, and cellular metabolism.^12^ This cerebral edema will ultimately culminate in increased intracranial pressure.

According to the Monroe-Kellie Doctrine, the total volume of the components of the intracranial compartment (brain parenchyma, cerebrospinal fluid and blood) remains constant since the skull is a rigid compartment incapable of expansion. When the normal intracranial volume is exceeded, as in the case of cerebral edema, intracranial pressure increases.^13^ Venous blood and cerebrospinal fluid can be compressed out of the compartment, causing pressure reduction, allowing intracranial pressure to remain normal shortly after the initial insult. Once the limit of displacement of cerebrospinal fluid and intravascular blood volume is reached, ICH occurs rapidly.^13^ In patients with a greater degree of cerebral atrophy, as occurs in adult cirrhotic patients, there is a greater quantity of cerebrospinal fluid available to be displaced, causing the increase in intracranial pressure to occur later.^1^ An increase in intracranial pressure consequently leads to a reduction in cerebral perfusion pressure (the difference between mean arterial pressure and intracranial pressure) and may cause cerebral ischemia.^13^ Thus, regardless of ICH, the presence of cerebral edema leads to a mechanism of secondary brain injury.^1^

Ammonia is produced primarily by intestinal bacteria, passing through the portal vein system and going to the liver, where it is detoxified to urea. In advanced liver disease, the loss of hepatocytes leads to insufficient detoxification through the urea cycle, leaving the detoxification of ammonia to glutamine synthetase present in the muscles and kidneys, which combines ammonia with glutamate to form glutamine.^1^^,^^14^ When the functional capacity of this enzyme in the muscles and kidneys is overwhelmed, serum ammonia levels begin to rise. Astrocytes are the only cells in the cerebral nervous system that have glutamine synthetase, leading to an accumulation of glutamine in these cells.^1^^,^^14^

Cytotoxic edema occurs due to the osmotic effects of glutamine on astrocytes, which attracts water into the cells, and due to the changes in cellular metabolism with alterations in the ionic gradient.^1^^,^^12^ Cytotoxic edema increases the concentration of ions and neurotransmitters in the interstitial space, which can reduce the seizure threshold and cause excitotoxicity. In addition, cytotoxic edema can dilute intracellular ions and the concentration of metabolites and disrupt metabolism.^12^ The role of Aquaporin 4 (Aqp-4) in the development of edema is also described. AQP-4 is a water channel present in the membranes of the astrocytes, close to ion channels (mainly potassium), and has the role of maintaining ionic balance.^1^ Ammonia has some properties like those of the ion potassium, able to pass through the channels and transporters of this ion, and to activate Aqp-4, as well as to increase its expression in response to the uptake of glutamine in the mitochondria, as described below.^1^

Vasogenic edema is a consequence of the breakdown of the blood-brain barrier with extravasation of plasma macromolecules, with increased intercellular oncotic pressure and movement of water into the brain tissue.^1^^,^^12^ This alteration of the barrier appears to be caused by the release of Metalloproteinase 9 (MMP-9) by necrotic liver cells, which leads to the degradation of occludin and claudin 5 and increased paracellular permeability.^1^^,^^12^

Cerebral edema initially was described as a feature of hepatic necrosis and then was established as a cardinal feature of ALF in the adult population, and it was believed not to occur in chronic liver disease. The incidence of cerebral edema in this condition and in ACLF has increased with the use of advanced techniques of magnetic resonance and computed tomography imaging studies as a method of identification of edema, not only by qualitative radiographic signs of edema or clinical signs of raised intracranial pressure.^1^ The degree of brain atrophy that can occur in chronic cirrhosis leads to a greater buffer against intracranial hypertension and can be an explanation for the less dramatic pattern of cerebral edema in chronic liver disease.^1^

Studies using magnetic resonance apparent diffusion coefficient and other technologies demonstrated that the pattern of edema present varies with the type of hepatic insult.^1^ In patients with acute liver failure, there is a predominance of cytotoxic edema, but vasogenic edema may also be present, initially localized in the basal ganglia, motor cortex, and cerebellum.^1^^,^^2^ In chronic liver disease, the main pathophysiological mechanism is vasogenic edema.^1^ Finally, in exacerbations of chronic cases, there is an overlap of cytotoxic edema, the extent of which is proportional to the severity of the acute insult.^1^^,^^2^

Although the role of glutamine and ammonia is still central to the pathophysiology of cerebral edema in both adults and children, new theories have been discussed as additional mechanisms of brain injury.^1^^,^^2^ This leads to the understanding that, depending on the phenotype of liver disease, the contribution of each individual mechanism may vary.^1^ Among these mechanisms, the most cited are the “Trojan Horse” and the glymphatic system and, despite the growing discussion about them, their clinical applicability is not well established, especially in the pediatric population.^14^, ^15^, ^16^, ^17^

A new hypothesis that has emerged is the “Trojan Horse” hypothesis.^15^^,^^16^ This hypothesis emphasizes the role of glutamine as a transporter of ammonia to astrocytes. Approximately 85% of the ammonia taken up by astrocytes is converted to glutamine by glutamine-synthetase.^1^^,^^3^ Thus, in patients with HE, there is a high production of glutamine, and the excess of this substance enters the mitochondria of the cells and is hydrolyzed by activated glutamine-phosphate, producing glutamate and ammonia. The increase in mitochondrial ammonia causes oxidative stress, collapse of mitochondrial oxidative phosphorylation, failure of ATP-dependent ion transport, and astrocyte edema.^1^^,^^3^

Another hypothesis close to the “Trojan Horse” hypothesis is the development of oxidative and nitrosative stress contributing to the formation of cerebral edema. It was seen that hyperammonemia reduces the activity of the antioxidant enzymes like glutathione peroxidase, superoxide dismutase, and catalase together with the formation of reactive oxygen and nitrogen species through a process associated with excessive activation of the N-Methyl-D-Aspartate (NMDA) receptor and intracellular accumulation of glutamine.^1^^,^^14^ In addition, these reactive species can lead to cerebral edema through activation of ion transport (oxidation of the Na/K/Cl cotransporter 1), activation of intracellular cascades, alteration of the blood-brain barrier by activation of metalloproteinases, and opening of transport pores in the mitochondria.^1^^,^^14^

Astrocytes are more glycolytic cells than neurons, and the lactate formed by glycolysis in astrocytes functions as an energy substrate for neurons.^1^^,^^17^^,^^18^ This lactate can be captured by neurons and converted to pyruvate in the Krebs cycle. In patients with liver dysfunction and hyperammonemia, ammonia will have an inhibitory effect on the Krebs cycle, causing impairment of cell energy metabolism. Thus, in these patients, there will be an increase in cerebral lactate with retention of high-energy phosphates, suggesting dysfunctional energy metabolism with frank energy failure until the final stages of HE.^1^^,^^17^^,^^18^

The involvement of the glymphatic system is one of the newest theories about the pathophysiology of cerebral edema in liver dysfunction. This system functions in a process in which the cerebrospinal fluid from the subarachnoid space passes through the perivascular space and thus circulates between the brain parenchyma, facilitating the clearance of toxic residues and metabolites from the interstitial space.^18^^,^^19^ It is possible that this mechanism is impaired in the setting of liver dysfunction, leading to the accumulation of toxins in the interstitial space, contributing to cytotoxic edema. Another hypothesis is that the impairment of fluid flow through this system may result in an increase in interstitial fluid, resembling vasogenic edema.^1^^,^^18^^,^^19^

In addition to these mechanisms that lead to edema, changes in the regulation of cerebral blood flow are also involved in the pathophysiology.^20^ CNS blood flow can be adjusted according to the brain’s metabolic activity, changes in perfusion pressure (pressure autoregulation), or changes in blood oxygen and carbon dioxide content. In patients with acute liver failure, a wide range of cerebral blood flow values has been described, ranging from very low to very high values; however, despite these variations, it is currently accepted that blood flow remains greater than the metabolic needs of the brain (luxury perfusion), with increased blood flow being a marker of a worse prognosis.^20^ The exact mechanism that leads to this hyperemia is not well established, but it appears to involve failure of the Na+/*K*+/ATPase pump and/or accumulation of certain substances such as cytokines, necrotic liver products, and glutamine, which lead to vasodilation of the microcirculation.^4^ There is also a loss of pressure autoregulation, which is shown by an increase in the mean velocity of the middle cerebral artery in response to an increase in mean arterial pressure.^20^

**Fig. 2:**
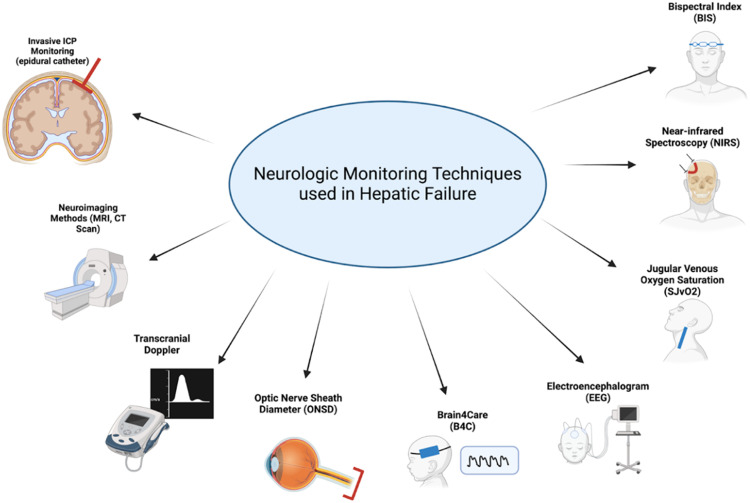
Neurologic monitoring techniques used in hepatic failure.

Taking these phenomena into account, neurologic monitoring techniques (Fig. 2) are an essential part of the critical care management of patients with liver failure. Below evidence is summarized to the most updated evidence regarding neuromonitoring, with a special consideration for non-invasive or minimally invasive techniques.

### Invasive ICP monitoring

Invasive monitoring is the gold standard for ICP management, allowing continuous real-time assessment of this parameter. It is performed by positioning a catheter in the epidural, subdural, ventricular space, or in the brain parenchyma.^3^^,^^4^ The objective of this monitoring is to maintain an ICP < 20 mmHg and adequate Cerebral Perfusion Pressure (CPP), considering metabolic and hemodynamic data.^4^ Sustained intracranial hypertension is present in up to 54% of adults with invasive monitoring and, despite the rationale that there would be an increase in survival due to better ICP management with this type of monitoring, this has not yet been extrapolated to clinical studies.^3^ On the other hand, a prolonged ICP > 40 mmHg and a CPP < 50 mmHg are associated with poor neurological recovery in patients with fulminant hepatitis who are candidates for transplant.^4^

In the guidelines aimed at the adult population, there are some indications for invasive ICP monitoring. According to the American Association for the Study of Liver Disease (AASLD), invasive ICP monitoring in patients with acute liver failure is indicated for those on the transplant waiting list and in specialized centers that use it.^21^ The European Association for the Study of Liver (EASL) suggests the use of invasive monitoring in patients with an increased risk of increased ICP, aiming for an ICP of 20‒25 mmHg and cerebral perfusion pressure of 50‒60 mmHg.^22^ Finally, the United States Acute Liver Failure Study Group (ALFSG) recommends the placement of an ICP catheter in patients with high-grade HE (III or IV).^4^ In general, it is acceptable for adult patients with HE grades III and IV to have this invasive monitoring, especially those awaiting liver transplant.^4^

Among the possible options for inserting the ICP monitoring catheter (intraventricular, subdural, epidural or brain parenchyma), the epidural is considered the safest method, despite having a lower accuracy in its reading than the other due to the presence of the dura mater. The intraparenchymal catheter, on the other hand, has a good correlation with the values obtained with the intraventricular catheters.^5^^,^^23^

The risk of bleeding has been described as 20% with epidural catheters and 22% with intraparenchymal catheters, with a fatal bleeding rate of 5% and 4%, respectively.^24^ A 2021 study in Canada evaluated the rates of bleeding complications in adult patients with acute liver failure and invasive ICP monitoring with an intraparenchymal catheter in a center with a well-established protocol for coagulopathy correction before invasive procedures. In this study, it was seen that 63.4% of patients had periprocedural hemorrhage, 53.7% with punctual subarachnoid bleeding considered not relevant and 28.6% with intraparenchymal hemorrhage, all asymptomatic and none requiring intervention.^25^

A 2015 retrospective cohort study of adults with acute liver failure evaluated whether invasive monitoring was associated with 21-day mortality, its association with therapeutic management, and its safety profile. A total of 629 patients with grade III and IV HE were evaluated, of whom 22% had invasive ICP monitoring, and this group received more therapies related to ICH and had a higher number of liver transplants despite similar mortality. Bleeding related to invasive monitoring was seen in 7% of the patients, and 5% of deaths were due to hemorrhagic complications.^26^ In a study in the United States, the bleeding rate in adult patients with grade IV HE and invasive monitoring was 4%, which corresponded to 1 patient who had an asymptomatic 5-mm subdural hematoma.^27^

Regarding the pediatric population, a retrospective review of patients under 18 years of age with acute liver failure and grade III and IV HE awaiting liver transplantation and using an ICP monitor with intraparenchymal catheters revealed that only 1 patient out of 14 (7%) had bleeding associated with catheter placement, developing subdural hematoma and an increase in ICP to 47 mmHg. Despite this complication, the patient did not show bleeding progression and was submitted to liver transplantation after 3 days and removal of the ICP catheter 7 days after its placement, without neurological dysfunction during follow-up.^9^

Currently, invasive ICP monitoring in children is reserved only when there is sufficient evidence from noninvasive methods that cerebral perfusion is inadequate. Evidence in the pediatric population is still very limited, and there are no studies showing that the insertion of an ICP catheter affects the prognosis of these patients.^2^ Most of the evidence on this monitoring method is based on studies with adults, and evidence in the pediatric population is still very limited; there are no studies showing that the insertion of an ICP catheter affects the outcome of these patients.^2^

#### Non-invasive neurological monitoring

Invasive ICP monitoring remains the gold standard for identifying increased ICP, but it is rarely performed in pediatrics due to its invasive nature, lack of expertise in the procedure, and increased risk of bleeding in the presence of severe coagulopathy.^10^ Therefore, alternative noninvasive methods are needed to evaluate ICP in these patients. Despite this, the evidence for these technologies in pediatrics is limited, lacking robust studies and randomized clinical trials. Table 1 below provides a summary comparing the different forms of neurological monitoring.

**Table 1:** Summary of the risks and benefits of different types of neurological monitoring techniques.

Neurologic monitoring	Benefits	Risks
Invasive icp monitoring	Gold-standard / Real-time assessment of the ICP	Bleeding / Differences in accuracy depending on catheter placement location
Neuroimaging	Exclude intracranial bleeding and differential diagnoses of HE	Findings with little correlation with ICP
Transcranial doppler	Noninvasive / Evaluation com cerebral autoregulation / Estimate the elevation of ICP / Monitor the response to treatment of ICH	Long period of practice for reliable results / Technical difficulties with difficulties to obtain an acoustic window
Optic nerve sheath diameter	Noninvasive / Correlation with ICP / Earlier changes than papilledema	Necessary training period and definition of cutoff value in pediatrics
Brain-4-care	Noninvasive / Correlation with ICP and alterations in intracranial compliance/Real-time assessment	Artifacts with patient movements / Lack of normative data on the P2/P1 ratio / Lack of an adequate device in patients with small head circumferences (< 34 cm)
Jugular venous oxygen saturation	Assesses cerebral metabolism and CNS oxygen consumption	Invasive / Lack of consensus on normal values / Risk of internal jugular vein thrombosis
Near-Infrared Spectroscopy (NIRS)	Noninvasive / Measuring regional SatO_2_	Correlation with ICP still remains uncertain / Lack of consensus on normal values

In pediatric patients, the differences when compared with the adult population regarding the natural history of chronic liver disease, in addition to the lack of adequate evidence in ACLF, argue against excluding these patients from consideration of noninvasive monitoring.^2^

## Neuroimaging

CNS imaging is necessary to exclude differential diagnoses of HE and identify findings of increased intracranial pressure. Cranial Tomography (CT) has a role in detecting intracranial bleeding, cerebral edema, compression of basal cisterns, hydrocephalus, midline deviation, and herniation, although it is not sensitive to identify increased ICP, and the absence of these findings does not exclude the presence of cerebral edema.^3^^,^^28^ In an analysis of patients with liver failure and HE grade III and IV managed with an ICP monitor, 84.6% of those with intraparenchymal catheters had ICH, and of these, only 30.8% had evidence of cerebral edema on pre-procedure CT.^29^

Cranial Magnetic Resonance Imaging (MRI) is more sensitive to detect cerebral edema, but it is more difficult to perform in critically ill patients on invasive ventilation. Findings of HE that can be seen on MRI include symmetrical and bilateral hyperintensity in the globus pallidus and substantia nigra on T1 and diffuse hyperintensity in the white matter of the corticospinal tract on T2/FLAIR.^3^^,^^4^

The diffusion method is a variant of MRI that is sensitive to local water diffusion restriction, providing quantitative information on water movement (Apparent Diffusion Coefficient ‒ ACD) and structural integrity (Fractional Anisotropy ‒ FA). This technique allows differentiation of changes in water distribution between the intracellular and extracellular compartments, with an increase in ACD being interpreted as an increase in the amount of water in the extracellular space, while a reduction is interpreted as an increase in the intracellular space.^30^

Despite all its usefulness, the findings of neuroimaging exams have little correlation with intracranial pressure.^3^^,^^4^

### Transcranial doppler

Transcranial Doppler is a method that has been studied and established in adults, with few high-level evidence studies in the pediatric population. This method evaluates the anterior, middle, and posterior cerebral arteries using an acoustic window at the temporomandibular junction with a Doppler wave.^3^ As in patients with liver failure, especially in the context of fulminant hepatitis, there is an impairment of cerebral autoregulation, which ends up leading to an alteration in the relationship between cerebral blood flow and ICP, as can be seen with a maintenance of the mean flow velocity through a variation in perfusion pressure.^2^^,^^11^^,^^28^ In addition to being a method used to estimate the elevation of ICP, Doppler can be used to monitor the response to treatment of ICH.^28^

The Pulsatility Index (PI) calculated by the Gosling method ([systolic - diastolic velocity]/mean velocity) has a good correlation with ICP, with an index > 1, associated to a reduction in the mean flow velocity, being related to a worsening of the neurological outcome as it was seen in a study that used this technology in children with severe traumatic head injury and correlated the PI and an increase in ICP above 20 mmHg in the first days after head trauma.^3^^,^^31^^,^^34^ Some studies have evaluated the calculation of Cerebral Perfusion Pressure (CPP) and ICP using cerebral artery velocities and blood pressure, with a good correlation between calculated ICP and invasively measured ICP.^32^ Despite these benefits, transcranial Doppler requires a long period of practice for reliable results, and the presence of technical difficulties with the inability to obtain an acoustic window in up to 20% of adult patients.^3^

In a 2019 case report of a 47-year-old patient with fulminant hepatitis who underwent liver transplantation, cerebral flow velocity was analyzed after the use of norepinephrine with the aim of increasing mean blood pressure by 20 mmHg.^11^ As a result, it was observed that cerebral blood flow velocity presented lower values before transplantation (despite the use of norepinephrine to increase mean arterial pressure by 20 mmHg), higher values immediately after transplantation, a tendency to normalize in 48‒72 h, and a new deterioration on the 5th postoperative day, coinciding with the time of diagnosis of hepatic artery thrombosis. These findings demonstrate an impairment of cerebral autoregulation in this patient.^11^ In general, in patients undergoing liver transplantation, a pattern of persistence of cerebral blood flow abnormalities is seen in the reperfusion phase, with subsequent normalization of autoregulation with a time interval until improvement of encephalopathy.^2^

Another study from 2012, performed in China, evaluated the feasibility of monitoring cerebral autoregulation with transcranial Doppler and Infrared Spectroscopy (NIRS) in 9 adult patients with chronic liver disease undergoing liver transplantation. The flow velocity of the middle cerebral artery was measured with transcranial Doppler and regional oxygen saturation with 2 sensors positioned on both sides of the frontal region connected to a NIRS monitor. The results demonstrate that impaired autoregulation and cerebral oximetry index are associated with higher MELD scores, and the mean velocity index in Doppler and the cerebral oximetry index present a good correlation (*p* = 0.0029) even in patients with bilirubin higher than 1.2 mg/dL.^33^

In a study that compared ultrasound methods and invasive monitoring of ICP, cerebral perfusion pressure, and ICP were estimated through values obtained in Doppler. The result of the study shows that PI is not accurate for detecting increased ICP, while the ICP estimated with the Doppler demonstrates a high Negative Predictive Value (NPV) for excluding increased ICP and may be used as a screening method for ICH.^32^

Few studies have evaluated this monitoring technique in the pediatric population, despite already being part of the monitoring protocol of some services.^2^ The first evidence on the subject was in the setting of severe traumatic brain injury, where a correlation was observed between increased PI and reduced diastolic velocity with an increase in ICP above 20 mmHg in the first days after head trauma.^34^ In 2022, the first study on the use of this technique in children in the setting of acute liver failure was published.^33^ It was a case series, carried out at the Bicêtre Hospital of the University of Paris-Saclay, that evaluated 10 patients with acute liver failure and severe HE who needed invasive mechanical ventilation and Continuous Renal Replacement Therapy (CRRT). The Doppler parameters evaluated were end-diastolic velocity and PI measured at the beginning of RRT and every 12‒24 h until 12 h after its discontinuation. The results showed a lower end-diastolic velocity in patients who died, with not one of the surviving patients losing End-Diastolic Velocity (EDV), a finding that was present in all patients who died.^35^

### Optic nerve sheath diameter (ONSD)

ONSD is a noninvasive method that uses ultrasound to estimate ICP by measuring the optic nerve sheath, a structure that is a continuation of the dura mater and has direct communication with the subarachnoid space, reflecting changes in cerebrospinal fluid pressure.^36^ The measurement is made by positioning the linear transducer across the eye, with the patient in the supine position and the head of the bed elevated at 30° The diameter of the optic nerve is then measured 3 mm posterior to the eyeball.^36^ Changes in this measurement occur earlier than the onset of papilledema.^3^ This measurement also has good inter-observer correlation^7^^,^^37^ and, unlike other methods, has a greater number of studies on its use in the pediatric population, but there is still a lack of larger studies and clinical trials on the subject.^2^

In 2019, a prospective Indian study was published that evaluated DBNO measurements in healthy children and children with acute liver failure and determined their correlation with the grading of HE. Forty-one patients were included, and the time to obtain the measurement in both eyes (in triplicate) ranged from 8 to 15 min. While DBNO was significantly higher in cases of acute liver failure with HE (5.2 mm [4.8‒5.8] mm) when compared with those without HE (4.4 [3.96‒4.65] mm; *p* ≤ 0.001) and those who had recovered from liver failure (3.9 [3.3‒4.1] mm; *p* = 0.02), there was no difference between patients without HE and with recovery from liver failure compared to healthy controls (4.2 [3.9‒4.3] mm; *p* = 0.1 and 0.2, respectively). Furthermore, a tendency for the ONSD to increase was observed according to the severity of HE, although it did not reach statistical significance. An ONSD value above 4.6 mm presented a sensitivity of 82% and a specificity of 87.5% for detecting HE, and above 4.9 mm, a sensitivity of 82.8% and a specificity of 73.3% for HE grades III and IV. In those who had clinical signs of ICH, there was a significant difference in the ONSD, which averaged 5.4 mm (*p* < 0.01). In predicting outcome, ONSD > 5.1 mm had a sensitivity and specificity of approximately 80% for worse outcomes in the absence of liver transplantation.^37^

Another Indian study, carried out in 2021, sought to define ONSD cutoff values in 31 pediatric patients (< 18-years) with acute liver failure and increased ICP. The study included 4 groups ‒ Group A: Presence of acute liver failure with clinical evidence of increased ICP (pupillary changes, bradycardia, and/or hypertension); Group B: Presence of acute liver failure without clinical evidence of increased ICP; Group C: Presence of acute hepatitis without signs of liver failure; Group D: Absence of liver disease. The mean ONSD value in children with acute liver failure and increased ICP was 5 mm, a value significantly higher than the ones found in the 3 other groups (*p* < 0.001). The ONSD values of groups B and C were also significantly higher than those of healthy controls (Group D). ONSD values greater than 4.55 mm at the time of diagnosis of increased ICP had a sensitivity of 87.5% and a specificity of 100% for identifying ICH. A significant reduction in ONSD was observed in those patients who responded to ICH management measures, with a reduction of 0.4 mm or more after 2 h of therapy, presenting a sensitivity of 84.3% and a specificity of 87.9% for ICH control. Finally, an ONSD greater than 4.6 mm after 24 h was a predictor of worse prognosis with a sensitivity of 83.3% and a specificity of 72.7% for survival assessment.^10^

In 2018, a study that included adolescents over 12 years of age evaluated ultrasound parameters (ONSD, estimated ICP, and PI) compared with an invasive method for detecting increased ICP.^33^ In this study, invasive monitoring was indicated for patients with a Glasgow Coma Scale (GCS) score of less than 9 and the presence of Grade IV HE. ONSD measurement in this population showed low sensitivity and specificity in detecting increased ICP and showed no association with increased mortality.^32^

Another fact observed is that in children with Grade I and II HE and absence of signs of ICH, the ONSD value was significantly higher than in children without neurological impairment, showing the presence of cerebral edema even in the earlier stages of encephalopathy.^10^ In addition, ONSD was shown to be related to INR and serum ammonia levels.^10^^,^^37^, ^38^

There are also studies that evaluate ONSD using cranial CT-scan and cranial MRI.^39^, ^42^^,^^43^ A case-control study, carried out in 2022 in an adult population, evaluated ONSD in patients with HE compared with controls without encephalopathy, demonstrating a significant relationship between increased ONSD and the presence of HE.^42^ In the same year, a Turkish study was published using cranial MRI to measure ONSD, with 40 patients aged 0 to 17 years with acute liver failure who underwent liver transplantation. The patients were divided into groups according to MRI findings: Group 1: No pathological intensity signal on T2W-FLAIR or diffusion; Group 2: Symmetrical involvement of the thalamus, posterior horn of the internal capsule, dorsum of the brainstem, periventricular or cerebellar white matter; Group 3: Diffuse cortical involvement. Groups 1 and 2 were classified as low risk and Group 3 as high risk for HE. ONSD values were associated with the groups of pathological findings on MRI (high risk ONSD 5.00 mm; low risk ONSD 6.48 mm; *p* < 0.001) but were not associated with the clinical grading of HE (*p* = 0.07).^43^

Regarding the cutoff value of ONSD, a study demonstrated that there is a progressive increase in its value with subsequent stabilization at 10-years of age, with cutoff values suggested as above 4.1 mm in children aged 4- to 10-years and greater than 4.4 mm in those over 10-years of age.^40^ In healthy patients aged 1- to 12-years in another study, the mean ONSD is approximately 4.5 mm, with values above this cutoff suggesting increased intracranial pressure.^41^ In children under 4-years of age, studies analyzing ONSD using computed tomography and magnetic resonance imaging indicate a cutoff value of 3.6–4.5 mm and 4.8–5.2 mm, respectively.^40^, ^41^, ^42^, ^43^ Other studies have shown a normal range in patients aged 5- to 9-years of less than 3.06 mm, 10- to 14-years of less than 3.17 mm, and 15- to 18-years of less than 3.37 mm.^10^ Cutoff values are summarized in Table 2.

**Table 2:** Evaluation and monitoring of pediatric patients with HE, according to its degree.

Periodicity	Hepatic encephalopathy Grade I‒II	Hepatic encephalopathy Grade III‒IV
Every 1h		Glasgow coma scale and pupillary assessment
Every 2h	Glasgow coma scale and pupillary assessment (until stable for 2 successive evaluations, then every 4-hours)	Vital signs
Every 4h	Vital signs	Capillary blood glucose
Every 6h		Metabolic panel and ammonia
Every 8h	Capillary blood glucose	
Every 12h	Metabolic panel, ammonia, blood count, liver function and transaminases	Blood count, liver function and transaminases
**Monitoring methods**		
Computed tomography	Always when grade III‒IV (upon diagnosis), focal signs, seizure and neurological deterioration.	
Optic nerve sheath diameter		Every 12-hours and when neurological deterioration
Transcranial Doppler		Once a day
Electroencephalography	Grade III‒IV, focal signs, seizure or neurological deterioration	
Brain-4-care (if available)		Every 6 h

Although there is a variation in normality values, a 2022 meta-analysis of DNOS in children stated that most studies used 4.5 mm or more as the ONSD cutoff value for differentiating raised and normal ICP and in studies with children younger than 1-year, ONSD cutoff values for differentiating raised and normal ICP were decreased, and 4.0 mm was used as the cutoff value. But, as they did not have raw data from the studies, a single ONSD cutoff value could not be established.^44^ In Table 3 below, the different cutoff values are summarized.

**Table 3:** Age-specific cutoff values for ONSD in the pediatric population.

Ages	Cutoff values
1-month ‒ 2-years-old (computed tomography)^42^	4.5‒4.0 mm
< 4-years-old (magnetic resonance)^43^	4.8‒5.2 mm
4‒10 years-old^40^	<4.1 mm
> 10-years-old^40^	<4.4 mm

### Brain4care® (B4C®)

Brain4care® is a technology created in 2006 by physics professor Sérgio Mascarenhas from the University of São Paulo after questioning the validity of the Monro-Kellie doctrine that the skull is a non-expandable compartment. This theory led him to discover a linear correlation between micro-expansions of the skull and changes in ICP. His student, Gustavo Frigieri, began a series of studies that allowed the conversion of these micro-expansions into ICP pulse curves that correlate well with invasive monitoring.^45^

ICP pulse curves are generated by the transmission of blood pressure from the choroid plexus to the cerebrospinal fluid and brain parenchyma. These curves have 3 components: P1, P2 and P3. P1 is called the percussion wave and represents arterial pulsation. P2 or the tidal wave indicates cerebral venous flow and is associated with intracranial compliance. P3 appears after the closure of the aortic valve, resulting in a brief interruption of blood flow and a drop in ICP.^45^^,^^46^ As ICP increases, there is a progressive exhaustion of compensatory mechanisms, leading to an increase in P2. The severity of ICH becomes even greater when P3 is greater than P1.^41^

As it is a relatively new technique, the use of B4C is still under study, and its evaluation in the HE scenario has not yet been carried out. A 2021 study in 6 adult ICUs compared parameters obtained by B4C and invasive intraventricular monitoring in neurocritical patients and demonstrated a better statistical correlation between the ICP waveform morphology of the two techniques and the ICP values themselves, showing that, despite having a good correlation, intracranial compliance and ICP may have different assessment rhythms. The P2/P1 ratio, when greater than 1.2 showed good accuracy in predicting ICP higher than 20 mmHg, especially in those with an intact skull.^46^ In 2022, the same group of researchers made the same comparison using B4C, but in patients with traumatic brain injury, also showed a significant relationship between the mean ICP measured invasively and the P2/P1 reaction, with a value greater than 1.2 having a sensitivity of 85% and a specificity of 77% for the presence of ICH. In addition, P2/P1 was significantly higher in those patients who died in the early post-trauma period.^47^

In the pediatric population, there are currently few studies on the use of B4C, two in the setting of hydrocephalus. The first is a 2017 study that evaluated ICP pulse wave patterns in children with hydrocephalus, which also demonstrated good sensitivity and specificity of P2/P1 for the presence of ICH.^48^ In 2021, a case report of a 7-year-old girl with idiopathic intracranial hypertension who underwent lumbar puncture along with ICP monitoring via B4C was published. It showed that before the puncture, the patient had a P2/P1 ratio of 1.1, followed by 1.38 during the procedure and 0.65 after the procedure.^49^ In 2024, an observational study carried out in Brazil was published in which the morphology of the intracranial pressure curve in healthy children was evaluated. The results showed an average P2/P1 ratio of 0.94 in the sitting position and 0.91 in the supine position.^50^ Despite these findings, there are no studies to date on the use of B4C in children with liver failure, and it is not known whether the data found on the use of the P2/P1 ratio can be extrapolated to this population.

B4C has two major limitations. First, the device is sensitive to patient movements and may generate artifacts hampering the measurements, an extremely important limitation in pediatrics. However, these artifacts can be reduced with the use of data processing algorithms. Second, there is a dependence on the operator to recognize the optimal acquisition of the curves since an inadequate position of the device may also alter the results. Another factor that must be considered is the lack of normative data on the P2/P1 ratio and peak time for healthy individuals of different age groups and sex, since the values obtained to date are based on critically ill patients. Additionally, there is a lack of an adequate device to accurately acquire data in patients with small head circumferences, generally below 34 cm (children < 1-year-old and congenital cranial malformations).^45^ More pediatric studies, including patients of different ages and pathological conditions, are needed for better validation.

### Jugular venous oxygen saturation (SJvO2)

This technique assesses cerebral metabolism and CNS oxygen consumption. For the measurement, a retrograde catheter is positioned in the internal jugular vein with the tip at the level of the venous bulb. Values considered normal are an SJvO_2_ of 50%‒70%, which corresponds to a cerebral extraction fraction of 30%‒50%. Another parameter that can be used is the arteriovenous oxygen difference (AVDO_2_ = CRMO_2_/CBF, where AVDO_2_ is the arterial-venous difference in Oxygen Saturation (SatO_2_), CRMO_2_ is the cerebral metabolic rate of O_2_ consumption, and CBF is the cerebral blood flow), and it reflects the amount of oxygen extracted from the brain. Under normal conditions, this value ranges from 2.2‒3.3 micromoL/mL.^3^^,^^4^

In situations of reduced perfusion pressure, there is an increase in extraction, leading to a drop in SJvO_2_ and an increase in AVDO_2_. Thus, reduced SJvO_2_ values may be justified by increased cerebral O_2_ utilization due to fever, seizures, or increased ICP with reduced perfusion. In situations of increased cerebral blood flow or reduced cerebral O_2_ utilization in cerebral edema, there will be an increase in SJvO_2_ and a reduction in AVDO_2_.^3^^,^^4^ In those with acute liver failure, persistent SJvO_2_ lower than 60% or higher than 80% is associated with elevated ICP.^30^^,^^31^ This method, however, does not detect changes in O_2_ consumption in small brain regions.^3^^,^^4^ The measurement of SJvO_2_ has some limitations, such as: lack of consensus on normal values and difficulty in comparing findings between studies, potential confounding effects of blood drainage from extracerebral structures, the fact that blood drainage from cortical structures is to the right jugular bulb and from subcortical structures to the left jugular bulb, risk of up to 40% of internal jugular vein thrombosis, among others. Thus, the measurement of SJvO_2_ allows inferences about cerebral metabolism but requires assessment of cerebral blood flow for its complete interpretation.^2^

### Near-Infrared Spectroscopy (NIRS)

A non-invasive method that works on the principle of the light absorption properties of hemoglobin, continuously measuring regional SatO_2_.^2^^,^^33^ In this technique, 4 wavelengths of the infrared spectrum penetrate the skull from a sensor, and the regional concentration of oxygenated and non-oxygenated hemoglobin is measured.^3^ NIRS does not differentiate between venous and arterial blood, and since most of the intracranial blood content is venous, cerebral oximetry allows a measurement of relative oxygen supply versus demand.^33^ Evidence regarding this monitoring device is mostly extrapolated from studies performed on the adult population.

In studies comparing findings in transcranial Doppler and NIRS, it was seen that the mean velocity index of the middle cerebral artery and the cerebral oximetry index have a good correlation in patients during liver transplantation.^33^

In the pediatric population, no general association was found between regional SatO_2_ and ICP in critically ill patients.^2^^,^^51^ In a study outside the context of liver failure, NIRS with cerebral SatO_2_ lower than 50% or higher than 80% was found to be associated with changes in head CT-scans of children with altered levels of consciousness.^52^ Although in some studies, performed in adult populations, NIRS has shown a correlation with the risk of neurological complications, its relationship with ICP still remains uncertain.^2^

### Continuous electroencephalogram (EEG)

EEG provides a functional assessment of the cerebral cortex, providing information on epileptic activity and the degree of sedation. If possible, performing the test during sleep and wakefulness increases the sensitivity of the method in detecting various alterations. It is useful in cases of acute liver failure, where an incidence of epileptic activity of 25%‒30% has been observed.^3^^,^^4^

Electroencephalographic alterations in hepatic encephalopathy can range from low-frequency alpha rhythm (8 Hz) mixed with bilateral theta activity, which can evolve with theta-delta waves with deceleration in both hemispheres, with or without triphasic curves. In severe coma, arrhythmic delta activity decreases, both in frequency and amplitude, progressing to cerebral silence.^3^

Subclinical seizures are rarely recognized in patients with grade III and IV hepatic encephalopathy, and EEG monitoring is important. In patients with fulminant hepatitis, cerebral ischemia precedes the onset of seizures, and in these patients, the extracellular concentration of glutamate predisposes to epileptic activity.^4^

In children with acute liver failure, changes in EEG waves of apparent prognostic significance have been described.^53^^,^^54^ These findings, although potentially valid, do not necessarily reflect intracerebral hemodynamics.^2^

### Bispectral index (BIS)

BIS is a recently described monitoring technique that allows the assessment of the effect of anesthetic drugs and their depth using EEG channels. This technique uses EEG of the frontal regions and incorporates the findings into an algorithm that transforms it into an adimensional number that indicates the patient's level of consciousness. Values range from 0 to 100, with 0 being complete suppression of electrical activity and 100 corresponding to the fully awakened patient. Values of 40‒60, during general anesthesia, have been shown to prevent awakening.^3^ Studies are needed to evaluate the role of BIS in monitoring the progression of HE, although this method may be useful for assessing the state of consciousness during the intensive care unit period and the pre-transplant moments in fulminant hepatitis, and the degree of recovery of the level of consciousness that accompanies the improvement of liver function after transplantation.^3^^,^^4^

### Practical management recommendations

Based on the concepts and techniques mentioned above, and taking into consideration practical aspects related to the availability of these monitoring techniques in the pediatric intensive care setting, mainly in medium-income countries, the group formulated a non-invasive approach to perform a serial evaluation of patients with HE. Recommendations are made based on the degree of HE and include clinical, laboratory and image studies (Table 2).

This recommended approach is currently being applied to patients in the studied institution, a quaternary pediatric transplantation center, part of a medical school hospital located in Brazil. The preference for the applied monitoring techniques and devices is related to their availability in the clinical environment. However, as mentioned above, many of the techniques are ubiquitously available in liver transplant centers.

## Conclusions

Hepatic encephalopathy and its progression to cerebral edema and intracranial hypertension are important causes of morbidity and mortality in patients with liver failure, whether acute, chronic, or ACLF. Therefore, adequate neurological monitoring of these patients is essential, especially for the early identification of neurological changes.

Although invasive ICP measurement is the gold standard, there is little evidence regarding its real benefit, in addition to the risks involved with the use of invasive devices, especially in patients with severe coagulopathy. Thus, the use of non-invasive forms of monitoring is gaining more strength, especially in the pediatric population.

In these patients, a multimodal neurological assessment is indicated, involving both serial clinical evaluation and a combination of non-invasive methods to ensure a better definition of the patient's neurological status and their risk of complications.

In the pediatric population, the authors currently have few robust studies and high levels of evidence regarding the use of various forms of neurological monitoring. Most of the evidence is based on studies in adults, with some points requiring evaluation regarding possible extrapolation to children. Therefore, new studies with a higher level of evidence in pediatrics are needed to establish the safest and most effective use of different forms of monitoring in this population. A possibility to surpass the challenge of a limited number of pediatric liver failure patients would be to arrange study networks including pediatric transplantation centers in different locations to promote studies regarding the various aspects of this somewhat neglected subgroup of patients.

## Data availability

The datasets generated and/or analyzed during the current study are available from the corresponding author upon reasonable request.

## CRediT authorship contribution statement

**Beatriz Kelly Oliveira Silva:** Conceptualization, Investigation, Writing – original draft, Writing – review & editing. **Maria Clara Silveira de Carvalho:** Writing – original draft, Writing – review & editing. **Artur Figueiredo Delgado:** Conceptualization, Supervision. **Werther Brunow de Carvalho:** Conceptualization, Supervision. **Michele Luglio:** Conceptualization, Investigation, Writing – review & editing, Supervision.

## Conflicts of interest

The authors declare no conflicts of interest.
